# Incidence of COVID-19 Symptom Rebound After Treatment with Remdesivir

**DOI:** 10.3390/idr17030043

**Published:** 2025-05-01

**Authors:** Kalpana Gupta, William J. O’Brien, Judith Strymish, Anna Chen, Katherine Linsenmeyer, Rebecca Madjarov, Michael E. Charness

**Affiliations:** 1 VA Boston Healthcare System, West Roxbury, MA 02132, USA; kalpana.gupta@va.gov (K.G.); william.obrien@va.gov (W.J.O.); judith.strymish@va.gov (J.S.); anna.chen2@va.gov (A.C.); katherine.linsenmeyer@va.gov (K.L.); rebecca.madjarov@va.gov (R.M.); 2 Department of Medicine, Boston University Chobanian & Avedisian School of Medicine, Boston, MA 02111, USA; 3 Veterans Affairs Informatics and Computing Infrastructure, George E. Wahlen Department of Veterans Affairs Medical Center, Salt Lake City, UT 84148, USA; 4 Department of Medicine, Harvard Medical School, Boston, MA 02115, USA; 5 Department of Neurology, Harvard Medical School, Boston, MA 02115, USA; 6 Department of Neurology, Boston University Chobanian & Avedisian School of Medicine, Boston, MA 02111, USA

**Keywords:** SARS-CoV-2, COVID-19, symptom rebound, remdesivir

## Abstract

Background/Objectives: Recent in vitro data suggest that remdesivir might be less likely than nirmatrelvir–ritonavir to be associated with COVID-19 rebound. We compared the incidence of symptom rebound in our remdesivir-treated cohort with rates reported in the literature for nirmatrelvir–ritonavir. Methods: We performed a retrospective cohort study of VA Boston Healthcare System patients who were nursing home residents or inpatients treated with remdesivir for mild to moderate COVID-19 that met clinical criteria for nirmatrelvir–ritonavir treatment between 05/2022 and 10/2024. Electronic health records were reviewed for evidence of symptom rebound in daily clinical evaluations and outside hospital care notes for 15–20 days after the diagnosis of COVID-19. Rates for nirmatrelvir–ritonavir were identified via a literature review. Results: Among 194 patients treated with remdesivir, 39 were excluded due to concurrent antiviral use, hypoxia, or ICU-level care. The average age of the remaining 155 patients was 75.1 ± 11.9 years; 147 patients (95%) were male. Evidence of symptom rebound was found in 1 of 155 (0.6%) remdesivir-treated patients, which is a rate lower than that reported in all 12 studies of nirmatrelvir–ritonavir symptom rebound during the Omicron era. Conclusions: Our finding of low rates of COVID-19 symptom rebound after treatment with remdesivir are consistent with the hypothesis that rebound may be less frequent after treatment with remdesivir than with nirmatrelvir–ritonavir.

## 1. Introduction

SARS-CoV-2 virologic and symptom rebound have been reported following treatment of COVID-19 with nirmatrelvir–ritonavir (N-R) [1,2,3,4,5,6]. The pathophysiology of rebound after antiviral treatment is uncertain, but mathematical modeling suggests that target cell preservation and delayed viral clearance play important roles [7,8,9,10,11,12]. Recent studies identified a persistent infectious, intracellular form of SARS-CoV-2 in permissive cells after exposure to the 3CL protease inhibitors nirmatrelvir and ensitrelvir [7]. The half-life for this infectious, intermediary form was approximately one day; in contrast, the SARS-CoV-2 polymerase inhibitor remdesivir eliminated intracellular infectious SARS-CoV-2 with a half-life of less than 6 h [7]. If persistent, intracellular infectious forms mediate SARS-CoV-2 rebound [7], then the incidence of rebound should be lower after treatment with remdesivir than with N-R. To test this hypothesis, we conducted a retrospective cohort study to evaluate the incidence of symptom rebound after treatment with remdesivir.

## 2. Materials and Methods

### 2.1. Patient Population

The study cohort was selected from all patients treated with remdesivir for mild to moderate COVID-19 between 5 May 2022 and 30 October 2024 in residential or acute care units at the VA Boston Healthcare System (VABHS). All eligible patients met criteria for outpatient treatment with N-R; however, their presence on residential or inpatient units and the potential for adverse drug interactions or the presence of kidney or liver disease made remdesivir a preferred antiviral to N-R. Patients residing in the community living center (CLC), a skilled nursing facility and nursing home, were included if they would have been eligible for outpatient oral therapy but received remdesivir because of their residence in a medically supervised unit. Patients requiring new supplemental oxygen, ICU-level care, or receiving N-R or molnupiravir were excluded.

### 2.2. Definition of Symptom Rebound

COVID-19 symptom rebound was defined as 48 h or more of increased COVID-19 symptoms (cough, shortness of breath, fever, weakness, hypotension, or respiratory distress), not otherwise explained, following at least 48 h of improved or resolved COVID-19 symptoms. A less stringent definition of symptom rebound was as at least one day of worsening COVID-19 symptoms, not otherwise explained, at least one day following a period of improvement.

### 2.3. Outcome Assessment and Follow-Up

Patients in residential or inpatient settings were evaluated multiple times daily by physicians, advanced practice professionals, registered nurses, nursing aides, and/or physical therapists. The electronic health record (EHR), including all nursing and provider notes, was reviewed from Day 0 through Day 20 for evidence of symptom rebound. For all patients who were discharged prior to Day 20, the EHR and community care notes were reviewed for post-discharge reports of symptom rebound. By hospital protocol, when a veteran receives care at an outside hospital, the event is captured and clinical notes are linked into the VA EHR, thus making available non-VA care information. CLC residents and inpatients were not specifically queried regarding the spectrum of COVID-19 rebound symptoms, although they were routinely asked about cough and shortness of breath. Suspected cases of symptom rebound identified through chart review were reviewed with an infectious disease specialist to determine whether the etiology was related to COVID-19 or COVID-19 rebound. Viral testing was conducted according to hospital protocols and was insufficiently frequent to identify virologic rebound.

### 2.4. Statistical Analysis

Our null hypothesis is that the incidence of symptom rebound does not differ between patients treated with remdesivir and those treated with N-R. Differences between the observed incidence of symptom rebound following remdesivir treatment and the reported incidence following treatment with N-R [2,3,4,5,6] were evaluated using bootstrap analysis. Because there is a wide range of reported incidence rates for symptom rebound in patients treated with N-R, we calculated 95% confidence intervals for symptom rebound incidence rates between 0.1% and 35% using cohort sizes of 155, our entire cohort, and 80, which was our most closely followed cohort. A simulation was conducted of 5000 instances of a binomial distribution with selected outcome probabilities and sample sizes. The mean outcome rates were calculated for the 5000 bootstrap samples, and, of those 5000 mean values, the 2.5th and 97.5th percentiles were calculated. Values outside the 2.5th and 97.5th percentiles were readily visualized, permitting a test of the null hypothesis across a range of incidence rates reported in the literature. 

This study was approved by the VABHS Institutional Review Board (protocol code 1789470-2; approved on 25 May 2024. The STROBE checklist for cohort studies was followed.

## 3. Results

Among 194 patients treated with remdesivir, 39 were excluded due to concurrent antiviral use, hypoxia, or ICU-level care. There were 155 eligible patients in the total cohort; their average age was 75.1 ± 11.9 years and 147 (95%) were male. Of these, 80 had at least 15 days of directly observed follow-up, with 70 (90.9%) having a full 20 days of follow-up. Their average age was not significantly different from the total cohort (Table 1).

Among the fully and directly observed follow-up cohort, 54 (67.5%) were residents of a VA community living center or spinal cord injury unit, 2 (2.5%) received care on inpatient mental health units, and 24 (30.0%) had prolonged inpatient stays on medical–surgical units for non-COVID primary diagnoses. The remaining 75 of 155 patients were directly observed for less than 15 days (average 6 ± 3.9 days); for those patients, evidence of symptom rebound was sought in both inpatient and outpatient notes in the EHR (Table 1).

Remdesivir was started on average less than one day after diagnosis and continued for a mean duration of 3.95 ± 1.6 days for the whole cohort. The majority of patients (91) received 3 days of therapy, 11 patients received 1–3 days of therapy, 35 patients received 4–5 days of therapy, and 18 patients received more than 5 days of therapy.

Evidence of symptom rebound was found in 0 of 80 (0.0%) of the full follow-up cohort and in 1 of 155 (0.6%) of the entire cohort, whether using the more stringent or less stringent definition of symptom rebound. The single patient who met the definition of symptom rebound had a liver transplant, was mildly symptomatic with cough and shortness of breath when initially diagnosed with COVID-19, and was able to be discharged from the hospital when these symptoms improved but then required readmission on Day 9 for recurrence of respiratory symptoms; he was re-treated with a second course of remdesivir without repeat viral testing.

In two patients, symptoms of rebound were attributed to other etiologies. One patient developed cough and shortness of breath on Day 16 and was diagnosed during an emergency room visit with an exacerbation of chronic obstructive pulmonary disease and pneumonia based on a left lower lobe infiltrate. A second patient with a history of bronchiectasis and pseudomonal pneumonia was readmitted on Day 13 after an initial response to remdesivir with new shortness of breath and an infiltrate consistent with bacterial pneumonia.

**Figure 1:**
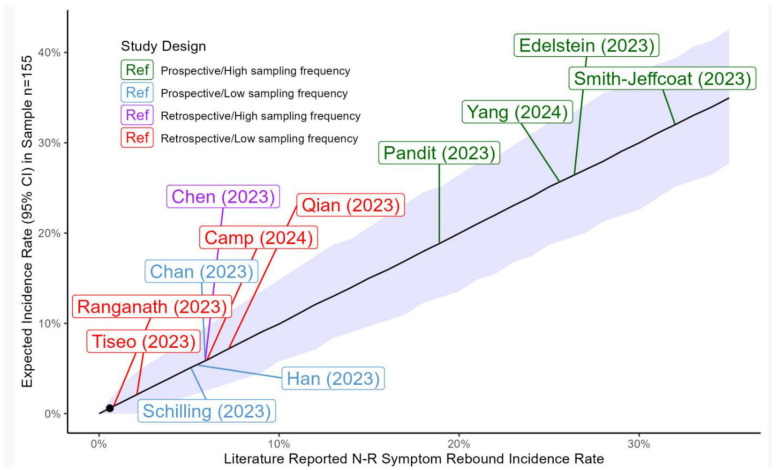
Confidence intervals for reported and expected incidence rates of symptom rebound. Shown are calculated 95% confidence intervals for symptom rebound incidence rates between 0.1% and 35% using a cohort size of 155 (Methods). Our observed incidence rate of 0.6% (black circle) is significantly lower than all published incidence rates greater than 3%. Incidence rates for symptom rebound in published studies of nirmatrelvir–ritonavir (N-R) are represented as colored boxes with citations referenced in the bibliography. Green: prospective studies with frequent sampling (at least every other day) throughout the expected period for rebound; blue: prospective studies with less frequent sampling; purple: retrospective studies with frequent sampling; red: retrospective studies with less frequent sampling.

Figure 1 shows the 95% confidence intervals for a wide range of expected incidence rates of symptom rebound modeled in cohorts with our sample size.

The observed incidence of symptom rebound (0.6%) in the entire cohort (*n* = 155) was lower than all incidence rates of symptom rebound reported after treatment with N-R during the Omicron era and significantly lower than all expected incidence rates greater than 3%. For the full-follow-up cohort (*n* = 80), the observed incidence of symptom rebound (0.0%) was significantly lower than all expected incidence rates greater than 5%.

## 4. Discussion

This observational cohort study of a real-world remdesivir-treated population supports the biological plausibility of low rebound risk recently proposed by in vitro work [7]. Our cohort of patients had mild to moderate COVID-19 and was demographically similar to outpatient populations treated with N-R; however, because they resided within our medical center, they were treated with remdesivir rather than N-R. Remdesivir treatment was initiated within one day of symptom onset, which is the initiation time period associated with the highest incidence of symptom rebound in N-R-treated patients described in multiple previous studies [2,8,9,10,11]. Frequent clinical monitoring of these patients during the expected onset of rebound, Days 10–12 [1,5,6], afforded an opportunity to observe rebound COVID-19 symptoms following treatment with remdesivir.

Our observed rate of symptom rebound following remdesivir treatment was significantly lower than most incidence rates reported after treatment with N-R during the Omicron era [2,3,5,6,13,14,15,16,17,18]. In prospective studies with high sampling frequency, rates of virologic and symptom rebound are higher in patients treated with N-R than in untreated patients [2,3,4,15]; however, rates range from 6% to 32% in N-R-treated patients because of differences in viral variants, population demographics, cohort ascertainment, treatment timing, definitions of rebound, and sampling frequency [2,3,4,14,19,20,21,22]. High sampling frequency is associated with higher detection of virologic and symptom rebound, whereas low sampling frequency fails to detect most cases [2,13]. The incidence of symptom rebound is also higher when a less stringent definition is employed, such as a single day of increased symptoms or a small increase in symptom score after symptom improvement [14,19,23]. Despite our high sampling frequency and even when we applied a less stringent definition of rebound, our observed incidence rate was comparatively low in patients treated with remdesivir.

Incidence rates of symptom rebound are lowest in retrospective cohort studies of outpatients that depend on identifying cases by reviewing the EHR [6,24]; these studies will miss cases when symptom rebound is not reported by patients or documented by providers. Our study should have detected more cases of rebound than these outpatient studies because most of our patients had in-person clinical evaluation several times daily.

Although specific COVID-19 symptoms were not elicited prospectively in our patients, general symptoms were assessed multiple times daily during routine clinical evaluation by providers and nurses who were aware of a recent COVID-19 diagnosis. Hence, the frequency of symptom ascertainment was even higher than in most published prospective studies of N-R symptom rebound, and the identification of clinically significant rebound symptoms should have been comparable if remdesivir and N-R were associated with similar incidence rates of symptom rebound. The low incidence rate of symptom rebound in our closely observed remdesivir-treated cohort is consistent with the observation that remdesivir rapidly eliminates intracellular, infectious forms of SARS-CoV-2 [7].

Other reports are consistent with our findings. In a study with low sampling frequency, Tiseo et al. [25] found symptom rebound in 0 of 196 outpatients treated with remdesivir and 2.1% of patients treated with N-R. The Federal Adverse Events Reporting System (FAERS) recorded strong signals for COVID-19 recurrence after treatment with N-R but not after treatment with remdesivir [26,27]. The observation that symptom rebound occurs at a similar rate in patients treated with N-R and VV116, a deuterated remdesivir derivative [14], may reflect a difference in pharmacokinetics of oral versus intravenously administered polymerase inhibitors [9].

This study has limitations. Our cohort was relatively small, disproportionately male, and selected from a single institution, thus limiting generalizability to other populations. Our study did not examine virologic rebound; therefore, some patients treated with remdesivir may have experienced asymptomatic virologic rebound [1,2]. Despite our high sampling frequency afforded by direct clinical observation by trained practitioners, the retrospective observational study design did not include formal ascertainment of COVID symptoms, and mild symptom rebound may have been missed. We did not have an N-R-treated group or an untreated group to provide a direct comparison with our remdesivir-treated group, though our observed incidence rate of symptom rebound after remdesivir treatment was significantly lower than rates reported after treatment with N-R in studies with similar or lower sampling frequency. Prospective studies with high sampling frequency are needed to better define the incidence rate of symptom and virologic rebound following treatment with remdesivir. Because virologic rebound may be associated with forward transmission of SARS-CoV-2 [1], confirmation of our findings would provide a rationale for selecting remdesivir to reduce the incidence of rebound and forward transmission in nursing homes and other congregate living settings.

## 5. Conclusions

Our finding of low rates of COVID-19 symptom rebound after treatment with remdesivir are consistent with the hypothesis that rebound may be less frequent after treatment with remdesivir than with nirmatrelvir–ritonavir.

## Figures and Tables

**Table 1:** Study cohort characteristics.

	Directly Observed for 15–20 Days	Directly Observed for <15 Days	Total Cohort
N	80	75	155
Age (years ± SD)	75.2 ± 11.6	75.0 ± 12.2	75.1 ± 11.9
Male	74 (93%)	73 (97%)	147 (95%)
Symptom rebound assessment through Day 20 of COVID diagnosis	Multiple nursing and clinician notes per day.	Multiple nursing and clinician notes per day until discharged.	
Symptom rebound assessment after discharge	N/A	Clinician notes from home-based primary care, repeated clinic visits, community care notes, and secure messages.	
Total directly observed follow-up (Days ± SD)	19.5 ± 1.4	6 ± 3.9	13 ± 7.4
Duration of remdesivir treatment	3.9 ± 1.5	3.9 ± 1.4	3.9 ± 1.4
Start time of remdesivir treatment from symptom onset	0.40 ± 0.6	0.44 ± 0.9	0.43 ± 0.7

## Data Availability

The datasets presented in this article are not readily available because of privacy restrictions. Requests to access the datasets need approval by local institutional research and privacy committees.

## Abbreviations

The following abbreviations are used in this manuscript:

ICU	Intensive care unit
EHR	Electronic health record
VA	Veterans Affairs
